# All in the (Definition of) Family: Transnational Parent–Child Relationships, Rights to Family Life, and Canadian Immigration Law

**DOI:** 10.1177/0192513X211054461

**Published:** 2021-12-05

**Authors:** Melissa Redmond, Beth Martin

**Affiliations:** Carleton School of Social Work, 6339 Carleton University, Ottawa, ON, Canada

**Keywords:** immigration/migration, Canada, parent–child relations, international adoption, human rights, definition of family

## Abstract

International human rights conventions, Canadian law and academic research all support the right to family life. Internationally and domestically, multiple definitions of family are recognized, acknowledging that long-term interpersonal commitments can be based on biological relationships as well as co-residential, legal, and emotional ties. Yet, the Canadian immigration system’s limited and exclusionary understanding of parent–child relationships complicates migrant family reunification. Drawing on qualitative interview and survey data from separated families and key informants who support them, we analyze national status and class assumptions embedded in Canadian immigration standards. We argue that Canadian immigration policies disproportionately deny the right to family life to transnational Canadians and their children who hail from the Global South and/or who are socio-economically disadvantaged. Immigration policies neither recognize the globally accepted “best interests of the child” welfare standard nor the human right to family life. We offer suggestions for addressing these inequities in practice and policy.

Sara and her husband had originally arrived in Canada as refugees fleeing political persecution. Once settled, they tried unsuccessfully, for many years, to have a child. When they realized that a family friend in their home country was looking for an adoptive family for her infant (who was in danger), all parties thought they had found a win-win solution. Sara and her husband returned briefly to their home country to adopt the child through the domestic courts, with the full consent of the birth mother. They had been advised that they would have no problem, as naturalized Canadian citizens, bringing the child back to Canada.

Unfortunately, as Sara went on to describe in her interview, the couple had been given incorrect advice. They subsequently discovered that Canada does not recognize any adoptions from their home country. As a result, Canadian immigration authorities refused the application, stating that the parents would “have to wait for the law to change.” Unable, due to continued persecution, to remain in their home country with their daughter, they were forced to leave her in the care of her adoptive grandmother.

At the time of the interview, they had been trying for 8 years to find a way to bring the child to Canada. With limited resources, they were unable to access lawyers, who may have been able to help with a petition on humanitarian and compassionate grounds. The situation had worn heavily on both Sara and her husband, who were increasingly fearful as their daughter approached teenage years in an unstable environment. Their daughter, who had only her elderly grandmother for support, was increasingly upset and confused about why she could not simply join her parents in Canada.

Sara and her husband were denied the right to live with their child. Their story exemplifies other research participants’ reports from a study by one of the authors. It investigated the experiences of immigrant families attempting to reunite in Canada. In that study, several participants described issues of ongoing separation due to proscribed definitions of family. These experiences stood out as overlooked and requiring of further examination.

The individual and family are basic components of society (Stewart, 2007). The fundamental nature of the family unit can be seen in globalized practices of individuals creating family, not only on the basis of biological relationships but also through long-term, supportive commitments that may include economic, co-residential, legal, or emotional ties (Braithwaite, 2010). Both the importance of and right to family are enshrined in numerous international human rights conventions (International Convention on the Rights of the Child, 1989; Universal Declaration of Human Rights, 1948), academic research (Simmons, 2008), and family law (Joly et al., 2016). Domestically, the Canadian state recognizes an increasing diversity of family forms. Yet the Canadian immigration system continues to be restricted to more limited, exclusionary notions of family.

There is little examination in the academic literature of this disconnect and its implications for families. Most family and migration literature considers families during the settlement process, after they have arrived together (e.g., Ali, Valade & Dargy, 2019) or once reunited following a period of separation (e.g., Suarez-Orozco et al., 2002). A large body of literature examines policies that govern international adoption by parents, usually from the Global North, who adopt children from the Global South. This research focusses on justified critiques of rich white Westerners adopting racialized children (Gibbons and Rotabi, 2012). Many of the studies on how policy shapes family migration are limited to cross-border spouses or common law partnerships (e.g., Gaucher, 2018; King-O’Riain, 2018). With the exception of Joly et al. (2016)’s work on the use of DNA tests in authenticating parental relationships, to date, there has been scant attention paid to the implications of the state’s understanding of parent–child relationships for immigration purposes. Few studies in the field have sought to examine the regulation of non-biological parent–child relationships between migrants and their children with common countries of origin.

In this study, we analyze how Canadian immigration policies enable or restrict migrants’ rights to parent–child relationships by examining contemporary human rights discourse, domestic practice, and the realities of migrant parents and their non-biological children from their country of origin. Using data collected from transnational families and those who support them, we examine the assumptions about families that are embedded in Canadian immigration regulations. Our findings show Canadian policies disproportionately curtail the right to family life of parents and children from the Global South and the socio-economically disadvantaged. We, further, argue that immigration practices often fail to recognize the globally accepted Best Interests of the Child (hereinafter BIOC) welfare standard.

We begin by reviewing literature on the global diversity and the importance of close relationships—often understood as *family*. We present the current policy framework, at an international and domestic level, and use transnationalism to examine the intersection thereof in Canadian immigration policy. Next, participant data illustrates a disconnect between the multiplicity of family forms recognized at international and domestic levels but not, at their confluence, in current immigration policy. We analyze the disproportionate and discriminatory implications for transnational families and point to the theoretical oversights exposed by the findings. We conclude with recommendations for research, practice, and policy.

## Literature Review

### Diverse forms of family

The myriad ways in which individuals gather in morally affirming and often economically supportive groupings are well-documented. People we are close to meet many of our basic needs, providing emotional and social support, a sense of belonging and security (Biehal, 2012). In this study, we define family as not only based in legal or biological connection, but also a result of the nature of relationship between individuals where a person assumes the rights associated with family *and* obligations implicit to such links (Stewart, 2007).

We argue that despite legal recognition domestically and internationally, certain family forms are privileged over others leading to explicit structural inequality (Statistics Canada, 2011; Tam et al., 2017a). In the Global North, the so-called *nuclear family*, of married heterosexual parents and their biological children in an economically self-sufficient unit, has too often rendered other forms of family invisible. Research has long supported the recognition of biological, structural, *and* functional definitions of family worldwide when considering the importance of family to individuals (Shah, 1998) and of integrating social and emotional interdependence outside the nuclear family into conceptualizations of family (Kağitçibaşi, 2006).

For example, the *functionally extended* family, where parent–child relationship quality is derived from family processes rather than concrete family structure is well-documented (Gonzales, 2007; Kağitçibaşi, 2006), with no discernible difference between adoptive, step- and biological parent families (Lansford, Ceballo, Abbey, & Stewart, 2001). In Indigenous communities in Canada, household membership complexities, multiple caregiver involvement and diverse naming and kinship conventions contrast with traditional Euro-Canadian familial notions (Tam et al., 2017b).

The *transactional approach* to family emphasizes self-identification—regardless of the existence of a biological relationship—of family members, who are often referred to as *fictive kin*. Examples of such family members include HIV/AIDS orphans in Africa raised by community members with whom they are not biologically related (Abebe & Aase, 2007); and an increasing trend in the Global North of friends taking on familial roles (e.g., Georgas, Berry & Kağitçibaşi, 2006). Voluntary family relationships have been shown to support the mental, physical and financial health of older individuals (Bedford & Blieszner, 2000); and to be extremely important to working class, queer, new immigrant, and street families (Ebaugh & Curry, 2000; Weston, 1991). Yet despite their popularity, diversity, and importance, *alternative families* are often considered incomplete, irregular or less legitimate (Nelson, 2014, p. 202). As others have noted, even the adjectives *fictive* and *chosen* serve to marginalize such relationships by conceptualizing them using a nuclear deficit comparison model (Weston, 1991).

### The importance of family

With positive outcomes for both the migrants and the host society, close and supportive family relationships are consistently shown to play an important role in migration experiences. For example, research has shown relatives who migrate as part of a family unit benefit from mutual emotional and social support as they settle in their new home (De Haene, Grietens & Verschueren, 2010; Nair, White, Roosa, & Zeiders, 2013). In turn, the act of supporting family members, especially dependent children, can provide migrants with a sense of purpose (Ali, Valade & Dargy, 2019). Migrating as a family unit allows for a certain cultural continuity, in contrast to the disruption often experienced by lone migrants (Rousseau, Mekki-Berada & Moreau, 2001).

Extensive research has documented the negative mental health consequences of family separation for both migrants (Letiecq, Grzywacz, Gray, & Eudave, 2014; Russo, Lewis, Joyce, Crockett & Luchters, 2015) and their family members who remain in home countries (Boccagni, 2013; Siriwardhana et al., 2015). For example, children may exhibit high levels of worry and fear of abandonment, and have problems academically or socially, when parental contact is limited (Moran Taylor, 2008; Rousseau et al., 2001). Relationships between parents and children can be severely impacted when separation is lengthy or indeterminate (Dreby, 2006; Martin, 2017a). Negative effects on the relationship can continue years after reunification (Boccagni, 2013). The well-documented importance of family for migrants and the diversity of family forms speaks to the need for inclusive definitions of family. The right to family has also been recognized by global legal conventions to which we now turn.

### A right to family life

At international (e.g., United Nations, UN) and regional (e.g., Americas) levels, there is widespread recognition of rights related to family. The Universal Declaration of Human Rights (1948) article 16.3, for example, describes family to be worthy of protection as the “natural and fundamental group unit of society.” No UN Convention or Covenant provides a clear definition of family, but the Office of the High Commissioner for Human Rights (2016) has advocated for a recognition of “various forms of family, depending on different cultural, political and social systems” (2016, p. 6). The same report centers the BIOC principle and urges non-discrimination in the protection of families in state application of international law.

The International Convention on the Rights of the Child (1989) states that a childs best interests should be a primary consideration in “all actions concerning children, whether undertaken by public or private social welfare institutions, courts of law, administrative authorities or legislative bodies” (Article 3). It also requires that “a child shall not be separated from his or her parents against their will” (Article 9). The specific rights of trans-border adoptee children are governed by the Convention of May 29, 1993 on Protection of Children and Co-operation in Respect of Intercountry Adoption, (hereinafter, the *Hague Convention*). It, too, emphasizes the importance of family life for children and adherence to the BIOC principle.

Regionally, the American Convention on Human Rights, 1969 recognizes the right of the family to protection by society and the state (Article 17) as well as the right of the child to measures of protection (Article 19). The Inter-American Commission on Human Rights (2013) has repeatedly highlighted the need for a wider-ranging definition of family life. They argue that the imposition of a narrow definition can be interpreted as interference in family life (17).

Canada is signatory to all the above conventions, except the *American Convention on Human Rights* (Canadian Civil Liberties Association, 2016). Canada also endorsed the non-binding *UN Global Compact on* United Nations Global Compact on Migration (2018), which urges states to support family reunification. In what follows, we compare Canada’s international commitments with the government’s domestic policy approach, highlighting an immigration policy disconnect.

Within Canada, non-biological, non-adoptive parent–child relationships can receive legal recognition. In the province of Ontario, for instance, *declarations of parentage* confer legal rights to adults who are neither adoptive nor biological parents. Multiple provincial court decisions have recognized the rights of adult platonic friends to co-parent, for the benefit of minor children (e.g., Bakht and Collins, 2018). According to the Children’s Law Reform Act. R.S.O (1990, c. C.12), decisions over child custody should consider the best interests of the child, which includes reference to “the love, affection and emotional ties between the child and the applicants” as well as the child’s wishes (24, 2).

Ultimately, the domestic child protection project recognizes parent–child relationships as mutable. A child’s best interests may warrant modification or dissolution of their relationship with their parents, thus permitting its replacement or supplementation with a caregiving relationship defined by characteristics other than biological parental ties. Thus, through judicial decision and policy implementation the Canadian state responds to the needs of its citizens in modifying its internal understandings of family. Yet when we turn to migrant families, we find that immigration policies serve to unduly restrict the right to family for certain migrants.

### Canadian immigration policies and the family

All permanent residency streams to Canada recognize the importance of family for migrants, through the inclusion of provisions for families to either migrate together or reunite in Canada. Indeed, each year, most new Canadian permanent residents are approved based on a close family relationship, whether as accompanying family members or an economic or refugee stream principal applicant, or as family class applicants sponsored by a relative already in Canada (Martin, 2019). The Immigration and Refugee Protection Act (2001) defines an eligible dependent child as a biological or adopted child aged 21 years or younger with the sole exception of an older adult child who is dependent due to mental or physical conditions. Evidence of a biological relationship between parent and child must be supported by official documentation. The state increasingly requests DNA certification as proof, particularly from African families (Joly et al., 2016).

The only non-biological parent–child relationships recognized by Canada for immigration purposes are adoptions that conform to specific criteria. International adoptions must follow the *Hague Convention* with approval from authorities, both in the parents’ home province and the country of the prospective adoptive child (Government of Canada, 2016). Adoptions must officially create a legal relationship between parent and child and extinguish previous parental-child legal bonds (Citizenship and Immigration Canada, 2015). Provincial and territorial authorities approve international adoptions only after completion of an in-depth application, an interview process called a home study, and extensive background checks (Ministry of Children, Community and Social Services, 2020). For those who adopt informally—a common occurrence in many countries—the validity of the parent–child relationship is often automatically impugned.

International adoption is not without critique. The exploitation by adoptive parents from the Global North of families in the Global South has been well-documented (Briggs, 2006). All Canadian jurisdictions have suspended approval of adoptions from several countries due to concerns over the reliability of their adoption systems (see Government of Canada, 2019 for the full list). Since 2013, the Canadian government has also prevented adoptions from certain Muslim countries. Reported in relation to Pakistan (Hill & Robinson, 2019; Nasser, 2018), Canada has claimed an incompatibility between its requirement [IRPR 3(2)] to sever the biological parent–child relationship, and an Islamic tradition that sanctions adoption where parental biological ties to a child should not be severed (Nasser, 2018). Known as kafala, this form of legal guardianship permits a child to receive care as part of another family while preventing the new guardians from *legally* supplanting the child’s biological parents. The Canadian government insists that *Hague Convention* commitments prevent it from continuing to facilitate adoptions in such cases. Yet, other Convention signatories, such as the United States and the United Kingdom, allow foreign nationals with adoption approvals from domestic courts to leave Pakistan and conclude adoption processes at home.

In summary, immigration policy includes more stringent and therefore, exclusionary, notions of family and requirements for verification than domestic policy. For non-biological parent–child relationships involving migrants, the possibility of being together is based on (a) the country of origin of the parents and children, (b) the timing of their relationship formation, and (c) whether (and how) the child was officially adopted.

## Theoretical framework

Next, we draw on the transnationalism literature to highlight the deep and disproportionate regulation through the Canadian immigration system of transnational parent–child relationships. Transnationalism theory reminds us that we cannot look at nations, nor migrants, as independent and isolated. It rejects previous migration theories and research that assumed discrete pre- and post-migration lives. Rather, transnationalism recognizes that migrants very often live lives that cross borders economically, socially, and emotionally, including transnational family life. Transnationalism allows us to raise questions about Canadian immigration systems and how we understand the organization of parent–child relationships.

The regulation of transnational lives is one of the ways in which immigration regimes allow the state to exercise “bio-power” (Foucault & Rabinow, 1978a, p.258) through practices, programs codes and policies that facilitate the “subjugation of bodies” (Foucault & Rabinow, 1978b, p. 308). The granting (or not) of entry into Canada is achieved through a surveillance regime, whereby individuals present themselves for examination—often before even reaching a physical border—hoping that they will meet the acceptance criteria. Thus, individuals are subject *to* the laws of Canada, even as not (yet) subjects *in* the law (Salter, 2006).

The love and emotional connection between interracial and international couples is subject to authentication; they must prove family status through performance and presenting “artifacts” of their love (D’Aoust, 2013, p. 264; see also King-O’Riain, 2018). Despite international human rights conventions, and domestic Canadian legislation acknowledging that family is not always defined by biological links or state-certified relationships, prescribed notions efface the emotive links between transnational couples unless enacted in a particular manner for immigration officials (Martin, 2017b).

In the rest of this article, we demonstrate how transnational parents and children are often not afforded the same opportunity to provide proof of their relationships. We highlight an oversight not only in the law, but also in the conceptual construction of the transnational families, that is, in the way we think theoretically about transnational families. By examining obscured narratives, we unveil a legal and theoretical centering of stereotypical white, heterosexual, middle-class prospective parents. This centering is at the expense of migrant parents and children from certain racialized countries who are rendered invisible.

## Methods

This article is based on the findings of the experiences of a subset of participants in a much larger project that was completed by the second author in 2017. The parent project was a mixed-methods analysis of the experiences of families trying to reunite in Canada through the family class stream of immigration. Following research ethics approval, data was collected through interviews and surveys from 169 families attempting to reunite, and 100 professionals (lawyers and consultants, settlement workers and constituency office caseworkers) who support applicants. Participants were located across Canada, with the sponsored family members representing over 50 different countries of origin.

With an ultimate goal of advocating for policy change, the study used a critical policy studies approach, drawing on intersectional and Foucauldian approaches to analysis (Hankivsky et al., 2014; Orsini and Smith, 2007). Analyses centered the stories of those who experience policy, to make visible processes and outcomes, particularly for certain marginalized groups, that have been “rendered invisible in traditional policy studies” (Phillips, 1996, p.251).

This article purposefully draws on data from a small sample of participants (with identifying details changed) who described a theme that has devastating implications for those who are affected and has yet to be investigated in academic literature. We used narrative analysis of two stories to investigate current immigration policies specific to international adoption and then integrated related data from key informants. Looking at those who were most affected by this issue also enabled us to problematize both how broader systems work to marginalize certain populations and query current theoretical, political, and administrative understandings.

## Findings

Two interview participants’ stories clearly reflected the devastating effect that the disconnect between definitions of family and Canadian immigration policy can have on individual families: an issue that settlement workers also flagged as critical. Sara’s story, with which this article opened, reflects the denial of an adoptive parent–child relationship based on domestic adoption in the country of origin. As we discuss later, this illustrates how Canadian implementation of the *Hague Convention*, designed to protect children, can work against the best interests of adoptive children of migrant parents.

Multiple key informants confirmed that Sara’s family was not alone and that they saw a lack of recognition for domestic adoptions for migrants from certain countries to be a recurring issue. Mahmaz, a settlement worker supporting primarily people from Muslim-majority countries, had seen this disconnect both through her work with migrants and in her own family:

The countries we are dealing with adoption is not legal here, you cannot adopt, but if they adopt from some countries which allow, […] one of my relatives she is there for like 9 years, she has adopted a daughter down there. She [the daughter] is family. She cannot bring the daughter.

Another survey respondent neatly summarized this form of denial of immigration applications:

The most common type of file I deal with that involves problems in the second stage is when a parent tries to sponsor an adopted family member, where the adoption was done as a private, domestic adoption. Many countries, such as China, will allow citizens of their country (who are not habitually resident in their country) to adopt family members through a domestic adoption without following the *Hague Convention*. Then, when the parent tries to sponsor the child, CIC (Citizenship and Immigration Canada) says the adoption is not valid because it didn’t follow the *Hague Convention*.

Other participants described the delegitimization of non-biological parent–child relationships that do not necessarily involve adoption. In his interview, Dieudonné explained how he had immigrated to Canada as a young adult. After several years, he was successfully established in a professional occupation and applied to sponsor his mother and younger sister, who was still a minor. As is not uncommon in their country of origin, his sister had been brought up by his mother from a very young age and had always been considered part of the family. However, when the Canadian authorities requested a DNA test and realized that mother and daughter were not biologically related, they demanded that Dieudonné remove his sister from the application.

He reluctantly did so, and his mother joined him in Canada, leaving her daughter with other relatives in the country of origin. However, at the time of the interview, Dieudonné reported that his mother found it too difficult to be separated from her (non-biological) daughter, with whom she was closer than Dieudonné, her (biological) son. She had decided to return to her country of origin to reunite with her daughter. The government’s failure to recognize his mother’s relationship with her daughter had resulted in indefinite separation for this family across continents. Dieudonné’s story illustrates the problematic reliance by Canadian authorities on DNA tests and a lack of recognition for links that domestically would be officially recognized as parental and familial relationships*.*

Mahmaz, a settlement worker, spoke with frustration about the impossibility for some families to meet Canadian government requirements for proof of family status:

Yeah, I have a case that is *er* she has adopted, actually she has picked up the child from the street like, it was like a financial assistance because the child was somebody, was born and put on the street and she, see, in not a very good condition. There’s no need. It was a war, this was zone, and this was work institution she has picked up the child. She has brought up the child and she has told the child for many years that he was adopted and now she’s having that son with her. But this was not Family Class, she was sponsored as a refugee, but she cannot keep, [she had to] leave that son behind. But there’s a problem: Whose child is this? Where are the parents? What documents do they have? How will they bring [the child] now?

Multiple key informants talked about clients who had taken on the parenting of minor relatives, usually nieces or nephews, who had lost their parents, or whose parents were unable to care for them, and the difficulties they faced when subsequently trying to bring those children to Canada. Vaina, a settlement worker, talked about how “sometimes they are making the mistake of claiming them as their own kids” not realising how the strict the Canadian immigration system is until a DNA test was requested, and the immigration authorities deemed the children ineligible. Lou, a lawyer, described the trauma experienced by a sponsoring applicant who was forced to revisit how she had taken over the care of her brother’s children and brought them up as her own after he had been murdered.

In all of these cases, the denial of family status not only affects the family members, but, as we discuss below, also contravenes the BIOC standard. These examples illustrate the disconnect that we would like to highlight between domestic and international recognition of the right to family life, and the denial of that right to certain transnational families. We turn to this next.

## Discussion

Our key message is noting the disconnect between the limited definition of family accepted in immigration policy and that recognized in international and domestic standards. Our findings show that Canadian immigration policy continues to deny the right to family life to certain families. Policies used to evaluate migrant families continue to be based on traditional and Western notions of family and fail to recognize legitimate family forms that are accepted by other Canadian regulatory bodies, domestically and at an international level.

Policies designed with the intention of protecting the best interests of children and preventing exploitation, marginalize Canadians who have transnational lives and fail to respect legitimate transnational family forms. They disproportionately deny the right to family life to Canadians and their children who hail from the Global South and/or who are socio-economically disadvantaged. Yet unlike domestic and international validation of family heterogeneity, familied migrants attempting to enter or reunite in Canada experience gatekeeping that curtails their attempts at family life.

The limited definition of family excludes long-standing pre-migration familial relationships such as those between Dieudonné, his sister, and their mother; relationships that have developed over a child’s lifetime despite lack of an official adoption. The restrictive understanding of parent–child relations also excludes families who *have* gone through the official adoption process in their country of origin, but who happen to hail from the wrong country, as with the case of Sara. Canada and other countries have justifiably introduced policies that address problematic adoption across borders by parents from the Global North of children from the Global South. However, the application of blanket moratoria on certain countries renders invisible migrants from those countries whose transnational lives include the formation of parental relationships with domestically adopted children.

As such examples illustrate, Canadians with transnational histories may find themselves in circumstances where they are unable to avail themselves of internationally recognized *or* local home-country rights. Such effects may arise because of pre-emptively narrow interpretations of family recognition mores or the lack of financial means to petition the government directly on humanitarian grounds. Domestic and international regulations, laws and practices would recognize similar parent–child relationships for Canadians born in Canada, but migrants’ access to family is unduly thwarted because of Canadian immigration policy’s pre-occupation with nuclear families and a blind allegiance to *Hague Convention* commitments.

As far as the Canadian systems that validate parent–child relationships (child protection authorities, immigrant officials) are concerned, all uncertified relationships and all adoptions involving certain countries are prima facie moot. At their core, the Western white nuclear foundations of these systems work in concert to effectively deny state bureaucratic support or legitimacy to Canadians born outside Canada. To be clear, the *Hague Convention*’s work in preventing the perils of child trafficking is laudable. To refuse automatically *all* adoptions from certain countries, however, fails to acknowledge legitimate parent–child relationships and leads to painfully indefinite separation. Nationals from countries that have been exploited by adoption shoppers are prevented from legitimating their adoptive relationships because *all* adoptions from such countries are now suspect. Often, this is despite the irrelevance for migrant parents of many of the concerns with international adoption cited by transnational adoption theorists (e.g.,ruptured connection to culture, language, and homeland) (Jacobson, 2008; Quiroz, 2012).

Transnational parents and their non-biological children face challenges of different evaluative standards relative to notions of kin. As Block (2015) argues, citizenship does not equal membership. Privileged Canadians can form state-sanctioned relationships with non-biological Canadian-born children. In direct contrast, non-Western family formations, regardless of citizenship status, are othered and face barriers to belonging (Horsti & Pellander, 2015).

By determining eligibility criteria and discounting the forms of evidence it will accept from applicants to evidence familial ties, the Canadian state applies “a normalizing gaze, a surveillance that makes it possible to qualify, to classify and to punish” (Foucault & Rabinow, 1977, p. 197). The result is that only Canadians who are socially powerful enough (more likely white, wealthy, and native-born) have a right to create family across borders. If the country they want to adopt from closes its borders to foreign adoption then—without a pre-existing national or cultural relationship to that country—they can move on. These options are not available to low-income transnational Canadians who *already have* transnational relationships with children in those home countries. Their rights to family are proscribed when they take on Canadian permanent residency and citizenship. The insistence on considering migrants solely in relation to their destination country renders their transnational lives invisible.

Ultimately, these policies have ongoing implications for the daily lives of parents and children in Canada and overseas. Canada is obliged to respect the BIOC standard, yet its immigration policy only recognizes the best interests of those children who have ties to certain Canadians. Family separation has considerable negative outcomes for children, their parents (like Sara), other members of their families (like Dieudonné), and their communities, both here in Canada and overseas. By prolonging parent–child separations, such policies contravene the very principle—BIOC— they were designed to uphold and instead can add considerably to suffering.

## Recommendations

We recommend recognition in literature, practice and policy of an expanded definition of legitimate family relationships. To date, international adoption research has considered Western/Northern choice for well-to-do-parents and subsequent foreign adaptation for transplanted children. The literature on non-biological parent–child relationships, in its limited focus on Western parents adopting internationally, has failed to expose the impact of regulatory systems on parent–child relationships that are transnational due to parental migration. The literature must overcome a pre-occupation with migrants’ liminal (bordered) experiences, to fully explore their lived transnational experiences of parent–child relationships. Thus, we invite more discussion regarding migrant families’ experiences as both objects of domestic immigration systems *and* as family-forming subjects objectified by those same systems.

Many professionals who work with transnational (or prospective transnational) families are well aware of the damaging effects of these conceptual gaps. Professional and ethical obligations render advocacy essential, to challenge current limitations to international adoption in Canadian immigration and adoption systems. Social service and immigration professionals can be an important part of bringing attention to these rights oversights, by petitioning provincial and federal governing bodies for regulations and practices that better reflect more consistently equitable standards of familial attribution and family reunification.

Continued advocacy is required for immigration systems to acknowledge the types of parent–child relationships that are recognized at a domestic level (e.g., fictive kin relationships). A recognition in application processes that not all familial bonds are biological or state-sanctioned would allow parents to petition governmental bodies using the same evidence of family life (e.g., photos, letters, financial records) as transnational couples. While the authors wholeheartedly support the protections and respect for children’s human rights enshrined in the *Hague Convention*, we offer that the current blanket bans efface the pre-migration lived experiences of many, including migrant parents and their children. For the only alternative to be the limited humanitarian and compassionate stream that requires significant resources to navigate is wholly discriminatory.

This study provides clear examples of the erasure of the lived experiences of certain naturalized Canadian nationals and their non-biological children, their transnational histories and associated rights. The findings explored many rights currently denied to transnational parents and children and challenge domestic and international immigration policies that work to undermine migrants’ right to family. The article started with Sara’s story and finishes with her request:

When they see people trying hard for something they should take the time to see the case you know this is a different case, they will see what we can do, not just deny it, putting it away, because that affect many, many lives […]. Seeing, denying a child to live with their parents in a better country.

I’m really glad, you know, I’m really glad with Canada because they open their arms to bring us here [as refugees] and we have lived a better life here, we’re not in risk as there. But that’s one point, that I encourage the government to take their time to see those cases and help them, right?
